# The Relationship between the Dietary Inflammatory Index (DII) and Metabolic Syndrome (MetS) in Middle-Aged and Elderly Individuals in the United States

**DOI:** 10.3390/nu15081857

**Published:** 2023-04-12

**Authors:** Qilong Zhao, Xinyue Tan, Zhenni Su, Habasi Patrick Manzi, Li Su, Zhenchuang Tang, Ying Zhang

**Affiliations:** 1 School of Public Health, Lanzhou University, Lanzhou 730000, China; 2 Institute of Food and Nutrition Development, Ministry of Agriculture and Rural Affairs, Beijing 100081, China

**Keywords:** dietary inflammatory index, metabolic syndrome, nutrition, diet, middle-aged and elderly people

## Abstract

(1) Background: With the aging demographic shift in society, there is a growing number of middle-aged and elderly individuals affected by metabolic syndrome (MetS), a risk factor contributing to all causes of mortality. Inflammation plays a crucial role in the development of MetS. This study aims to examine the correlation between MetS and pro-inflammatory diets in middle-aged and elderly individuals, utilizing the Dietary Inflammation Index (DII) as a measure. (2) Methods: Data were extracted from the 2007–2016 National Health and Nutrition Examination Survey (NHANES) database for individuals who were 45 years of age or older. The DII was determined for each participant through 24-h dietary recall interviews. The relationship between DII and MetS was assessed using binary logistic regression analysis, and the association between DII and MetS-related indicators was further explored through generalized linear model (GLM) and quantile regression analysis. (3) Results: A total of 3843 middle-aged and elderly individuals were included in the study. After controlling for confounding factors, the highest quartile of DII was associated with a higher risk of MetS (OR${}_{Q4:Q1}$ = 1.339; 95% CI: 1.013, 1.769; *p* for trend = 0.018). The top DII quartile also increased the risk of reduced HDL-C (OR${}_{Q4:Q1}$ = 1.499; 95% CI: 1.005, 2.234; *p* for trend = 0.048) and raised FG (OR${}_{Q4:Q1}$ = 1.432; 95% CI: 1.095, 1.873; *p* for trend = 0.010) compared to the lowest DII quartile. The levels of DII were found to be positively correlated with BMI (β = 0.258, *p* = 0.001), FPG (β = 0.019, *p* = 0.049), TG (β = 2.043, *p* = 0.013), waistline (β = 0.580, *p* = 0.002), and negatively correlated with HDL-C (β = −0.672, *p* = 0.003). (4) Conclusions: In middle-aged and elderly individuals in the United States, a high DII score has been linked to the presence of MetS, low HDL-C, and hyperglycemia. Therefore, dietary recommendations for the middle-aged and elderly should focus on reducing the DII by choosing foods rich in antioxidants, dietary fiber, and unsaturated fatty acids.

## 1. Introduction

Metabolic syndrome (MetS) is a condition characterized by central obesity, high blood pressure, high blood sugar, and abnormal lipid levels, which is closely associated with cardiovascular disease, diabetes, and fatty liver disease [1]. With advancements in socioeconomic conditions and alterations in human lifestyles, the occurrence of MetS is on the rise and has become one of the most pressing health concerns in modern society [2]. The incidence of MetS globally is continuously increasing, with an estimated one-third to two-thirds of adults suffering from it [3]. A meta-analysis of 15 representative countries in the Asia-Pacific region also found that the prevalence of MetS is as high as 20% and continues to increase annually [4]. The U.S. Centers for Disease Control and Prevention reported in 2017 that approximately one-third of Americans are affected by MetS [5]. According to the World Health Organization (WHO), the prevalence of metabolic syndrome in middle-aged and elderly individuals ranges from 11% to 43% (median 21%), while the National Cholesterol Education Program (NCEP) reports it to be between 23% to 55% (median 31%) [6,7]. Furthermore, MetS in middle-aged and elderly individuals is a substantial risk factor for cardiovascular morbidity and mortality [8]. Therefore, there is an urgent need for preventative measures to reduce the incidence of MetS among middle-aged and elderly individuals.

The inflammatory response is a multifaceted biological process that involves several cytokines, such as interleukin-6 (IL-6), tumor necrosis factor-α (TNF-α), and C-reactive protein (CRP). If this process becomes dysregulated, it can result in chronic inflammation and tissue damage [9]. Current research demonstrates that MetS arises from chronic low-grade inflammation in the body, which is strongly linked to the incidence of both diabetes and hypertension [10,11,12]. Extensive studies have highlighted the significant impact of diet on the body’s inflammatory response and have demonstrated that inadequate dietary habits can contribute to the development of MetS [13,14,15,16]. For example, dietary components such as unsaturated fatty acids, fiber, and vitamins have been shown to reduce body inflammation and lower the risk of MetS, while supersaturated fatty acids, high cholesterol, and high carbohydrate intake may contribute to inflammation and increase the likelihood of MetS [17]. As a result, Examining the risk factors for MetS can be achieved by considering the inflammatory effects of one’s diet. The Dietary Inflammatory Index (DII), which was created by Shivappa et al. in 2009 and refined through a scoring system in 2013, is a credible measure of the extent to which dietary factors contribute to an individual’s inflammatory response [18]. Currently, DII has been increasingly employed in the fields of nutrition and epidemiological research as a method of evaluating an individual’s dietary-related inflammation. In this matter, numerous studies have assessed the correlation between MetS and DII in various demographic groups. A positive association has been observed in Spain between DII and body fat percentage, waist circumference, and body mass index (BMI) among individuals with an increased risk of cardiovascular disease [19]. Research on police officers has revealed that those with a higher DII are more likely to have MetS [20]. A prospective study in France on the SU.VI.MAX population showed that the DII is positively correlated with the risk of MetS [21]. The same results were still found in studies in Iran and South Korea [22,23].

While previous studies have shown the correlation between DII and MetS in both general and specific populations, research on middle-aged and elderly groups is limited. Additionally, some studies have failed to establish a definitive link between DII and MetS [24,25]. The decline in basal metabolic rate (BMR) that commonly occurs in middle-aged and older adults, in combination with inadequate physical activity, weakened immune system function, and prolonged medication use, increases the risk of developing MetS in this population [26,27]. Furthermore, with the aging of the American population, it is estimated that nearly 50% of individuals over 60 years of age have MetS, making it a major challenge for public health care systems [28]. Thus, it is important to evaluate the relationship between DII and MetS in elderly Americans and the relationship between specific components of MetS.

## 2. Materials and Methods

### 2.1. Data Sources

The data utilized in this study were acquired from the National Health and Nutrition Examination Survey (NHANES), which was accessed for free on the official NHANES website. The NHANES survey is conducted biannually by the Centers for Disease Control and Prevention (CDC) and employs multi-stage complex sampling to ensure a representative sample [29].

### 2.2. Study Population

**Figure 1:**
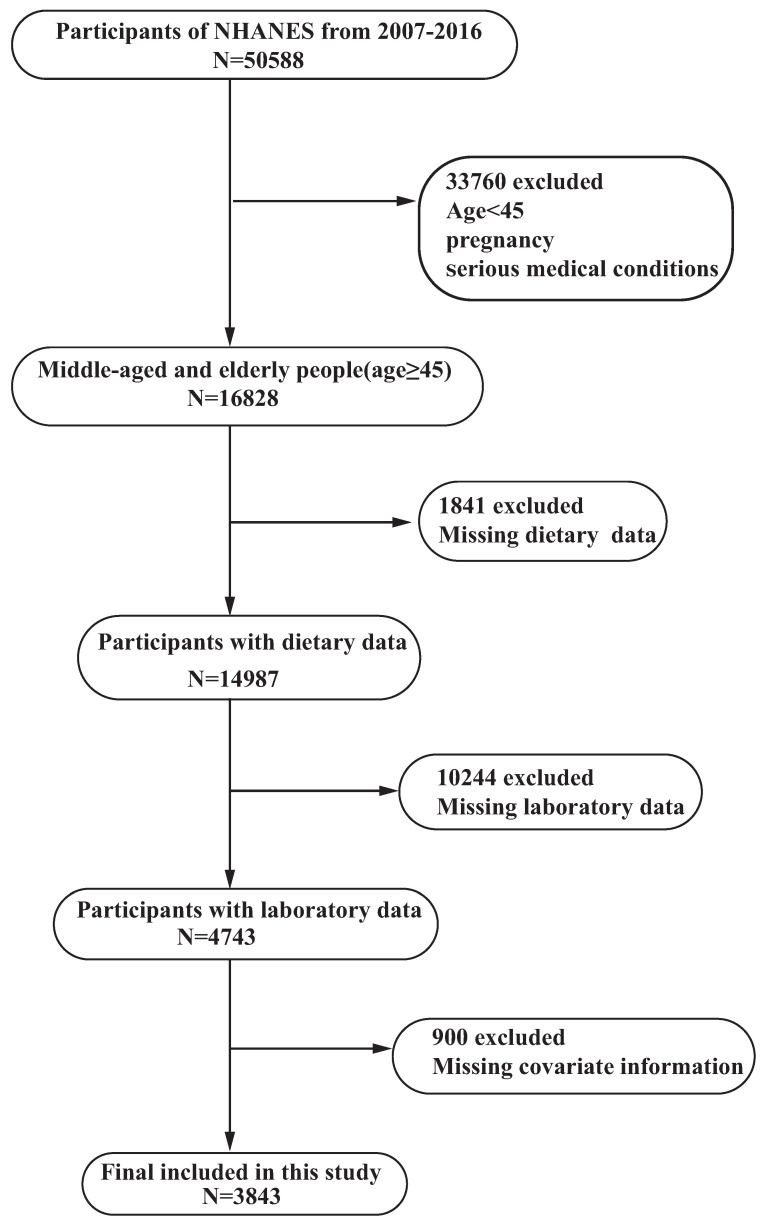
Flow diagram of participants in the study.

The study sample was selected based on the following criteria: inclusion criteria included all samples aged ≥45 years in the NHANES database from 2007–2016, while exclusion criteria were (1) pregnant women, (2) participants with serious diseases (e.g., malignancy, coronary heart disease, etc.), and (3) participants with missing data on key indicators such as demographic, laboratory and questionnaire data, and dietary data. A total of 3843 study subjects were included after applying these criteria. (Figure 1 and supplementary file).

### 2.3. Measurement

#### 2.3.1. Definition of MetS

According to the National Cholesterol Education Program (NCEP) guidelines from 2005, MetS is defined as having three or more of the following characteristics [5,30]: (1) Raised fasting blood glucose (Raised FG), fasting blood glucose >5.6 mmol/L (100 mg/dL) or drug treatment for raised blood glucose; (2) Reduced HDL cholesterol (Reduced HDL-C), HDL cholesterol <1.3 mmol/L (50 mg/dL) in women, <1.0 mmol/L (40 mg/dL) in men or drug treatment for reduced HDL cholesterol; (3) Raised blood triglycerides (Raised TG), blood triglycerides >1.7 mmol/L (150 mg/dL) or drug treatment for raised triglycerides; (4) Raised waist circumference (Raised WC), waist circumference >88 cm (women) or >102 cm (men); (5) Raised blood pressure (Raised BP), blood pressure >130/85 mmHg or drug treatment for raised blood pressure.

#### 2.3.2. Dietary Inflammation Index Calculation

The dietary database’s nutrient information of the first day was selected as the accurate nutrient intake for each participant in the study. A total of 27 nutrients, including total energy, fat, saturated fat, monounsaturated fat, polyunsaturated fat, ALA (octadecatrienoic acid), EPA (eicosapentaenoic acid), DHA (docosahexaenoic acid), docosapentaenoic acid, linoleic acid (octadecadienoic acid), arachidonic acid (eicosatetraenoic acid), protein, carbohydrates, fiber, alcohol, cholesterol, niacin, vitamin (A, B${}_{1}$, B${}_{2}$, B${}_{6}$, B${}_{12}$, C, D, E), iron, zinc, selenium, magnesium, folic acid, beta carotene, and caffeine were considered in the calculation of the DII. The *n*-3 fatty acids were equal to the sum of ALA, EPA, DHA, and docosapentaenoic acid, and the *n*-6 fatty acids were equal to the sum of linoleic acid and arachidonic acid.

The average and variability of each nutrient were obtained from the World Diet Standards Library and used to transform the Z-scores of the respective nutrient into Z-transformed scores. The Z-transform fraction of each nutrient was then transformed into percentiles, and the resulting distribution for each nutrient level was made symmetrical around 0 (zero) by doubling the transformed percentiles and subtracting 1. The bounds of this distribution are −1 (maximum anti-inflammatory) and +1 (maximum pro-inflammatory). The final DII score is obtained by multiplying each nutrient level by its respective corresponding inflammatory fraction and summing the result [18].

#### 2.3.3. Other Covariates

Covariates considered in the analysis included sociodemographic factors such as gender (male/female), age (middle-aged: 45 to 60, elderly: over 60), race (Hispanic White, Non-Hispanic White, Mexican American, Non-Hispanic Black, and other races), education (less than 9th grade, 9th to 11th grade, high school graduate/GED or equivalent, some college or AA degree, college graduate or higher), and poverty level expressed as poverty–income ratio (PIR) (PIR ≤ 1, 1 < PIR ≤ 1.99, 2 < PIR ≤ 3.99, and PIR > 4). In addition, behavioral factors including smoking status (defined as smoking more than 100 cigarettes, with all others considered non-smokers), alcohol consumption (defined as having more than 12 drinks in the past year, with all others considered non-drinkers), and sedentary behavior (total sedentary time of less than 3 h, 3 to 5.9 h, and 6 h or more) were considered.

### 2.4. Statistical Analysis Methods

Data description and statistical analysis in this study utilized complex weighting through the use of the “survey” package in R software. Categorical variables were described by their frequency and proportion, and differences were tested using the Rao–Scott $\chi^{2}$ test. Quantitative data that were not normally distributed were described as median (25th percentile, 75th percentile), and group comparisons were performed using the Wilcoxon rank sum test. A binary logistic regression model was employed to examine the relationship between quartile groupings of the DII and MetS and its components, and the median of each quartile grouping of DII was used to complete the linear trend test associated with the disease. To explore potential differences based on gender and age, separate analyses were conducted for gender (males and females) and age (45∼60 years and over 60 years). The association between DII levels and the risk of MetS and its components was further explored through restricted cubic spline analysis with four knots at the 5th, 35th, 65th, and 95th percentiles of its distribution. A generalized linear model was employed to evaluate the relationship between DII levels and biochemical indicators of MetS, while quantile regression models were utilized to assess the strength of the relationship between DII levels and MetS-related parameters at different quantile points of each indicator.

Potential confounding variables were taken into account throughout the analysis, including gender (male or female), age (45∼60 or over 60), race (Hispanic White, Non-Hispanic White, Non-Hispanic Black, Mexican American, and other races), education (less than 9th grade, 9∼11th grade, high school graduate/GED, some college or AA degree, or college graduate or higher), poverty–income ratio (PIR less than or equal to 1, 1 less than PIR less than or equal to 1.99, 2 less than PIR less than or equal to 3.99, or PIR greater than 4), smoking (yes or no), drinking (yes or no), and sedentary behavior (total sedentary time of less than 3 h, 3 to 5.9 h, or 6 h or more). The analysis was conducted using R 4.2.0, and a *p*-value less than 0.05 was considered statistically significant.

## 3. Results

### 3.1. The DII Quartile Feature Distribution of the Study Object

The DII scores range from −5.20 to 4.66, with negative scores indicating anti-inflammatory diets and positive scores indicating pro-inflammatory diets. The first quartile, which ranges from −5.20 to −1.00, represents the group with the most anti-inflammatory diets. The second quartile, which ranges from −1.00 to 0.63, represents the group with moderately anti-inflammatory diets. The third quartile, which ranges from 0.63 to 2.05, represents the group with moderately pro-inflammatory diets. The fourth quartile, which ranges from 2.05 to 4.66, represents the group with the most pro-inflammatory diets.

Table 1 presents the distribution of the characteristics of the study participants based on their quartile of the DII score. Variables including age, smoking habits, sedentary behavior, TC, LDL-C, HDL-C, FPG, and waist circumference showed significant differences in their distribution among the DII quartile groups. The proportion of women increased progressively from a minimum of 50.9% in the Q1 group to the Q4 group, while the proportion of men declined from 38.34% in the same group. The proportion of individuals with a college degree or higher education decreased in the DII grouping, with the highest proportion of high school graduates or equivalent being in the Q4 group. The distribution of non-Hispanic black people in the DII group was most pronounced. Participants with a poverty–income ratio (PIR) less than 2 showed an increasing trend in the DII grouping, while those with a PIR greater than 2 showed the opposite. The proportion of individuals with the MetS also increased, with 38.25% of the Q4 group being affected. Furthermore, participants in the Q4 group had higher levels of SBP, DBP, TG, OGTT, and BMI values.

### 3.2. Association of Dietary Inflammatory Levels with MetS

**Figure 2:**
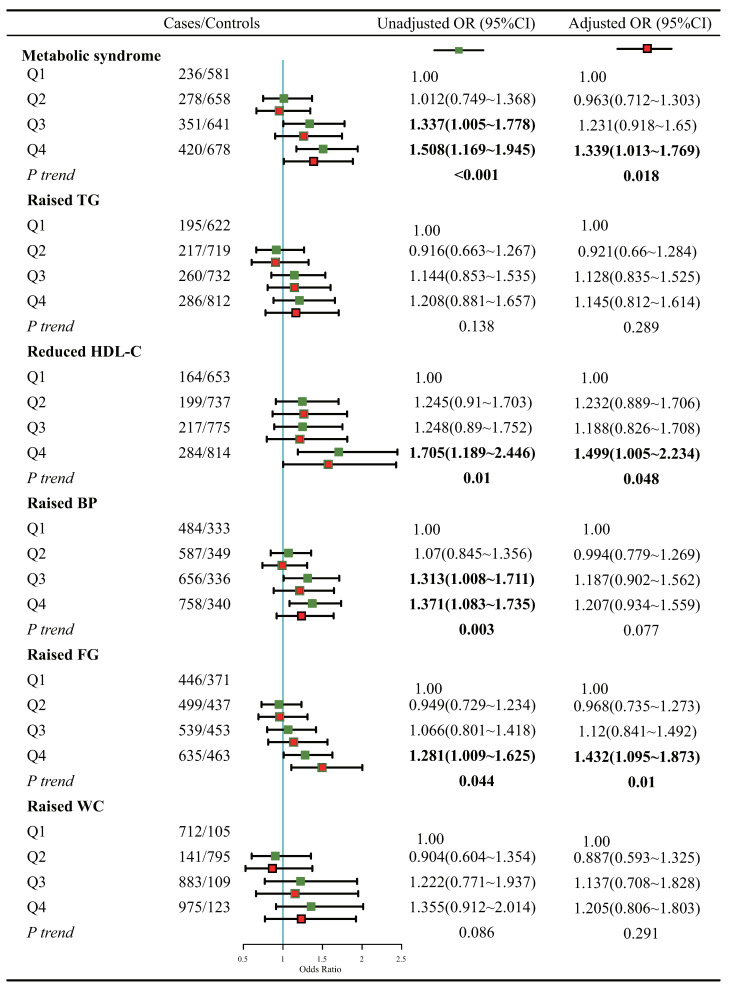
Association of DII with MetS and its components. Adjusted for confounding factors such as age, gender, race, education, poverty–income ratio, smoking, alcohol consumption, and sedentary behavior.

**Figure 3:**
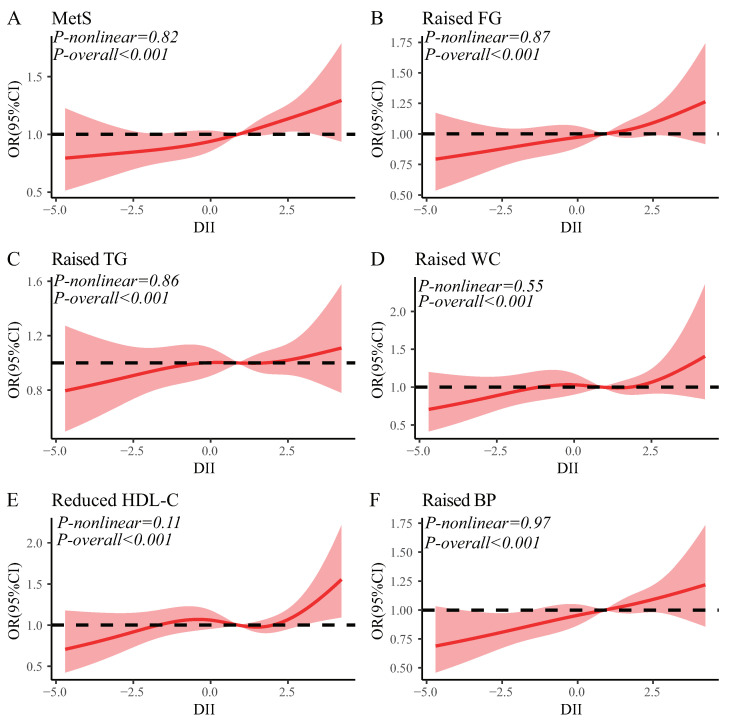
The restricted cubic spline for the associations of DII with MetS and its components. (**A**) Metabolic syndrome; (**B**) Raised FG; (**C**) Raused TG; (**D**) Raised WC; (**E**) Reduced HDL-C; (**F**) Raised BP. Knots were placed at the 5th, 35th, 65th, and 95th percentiles of the DII distribution. Results were adjusted for age, gender, race, education, poverty–income ratio, smoking, alcohol consumption, and sedentary behavior.

The binary logistic regression model was employed to evaluate the correlation between DII and the presence of MetS as a whole, as well as its individual components (Figure 2). After controlling for sociodemographic factors and behavioral information, the group with high DII(Q4) was found to increase the risk of MetS by 1.339 times (OR${}_{Q4:Q1}$ = 1.339, 95% CI: 1.013, 1.769, *p*-trend = 0.018), compared to the group with the lowest DII (Q1). When considering the components of MetS individually, the results showed that DII was statistically significant in its association with reduced HDL-C (OR${}_{Q4:Q1}$ = 1.499, 95% CI: 1.005, 2.234, *p*-trend = 0.048) and raised FG (OR${}_{Q4:Q1}$ = 1.432, 95% CI: 1.095, 1.873, *p*-trend = 0.010). Furthermore, the spline regression models demonstrated a significant increase in the risk of both MetS and its individual components with increasing DII scores (Figure 3).

**Figure 4:**
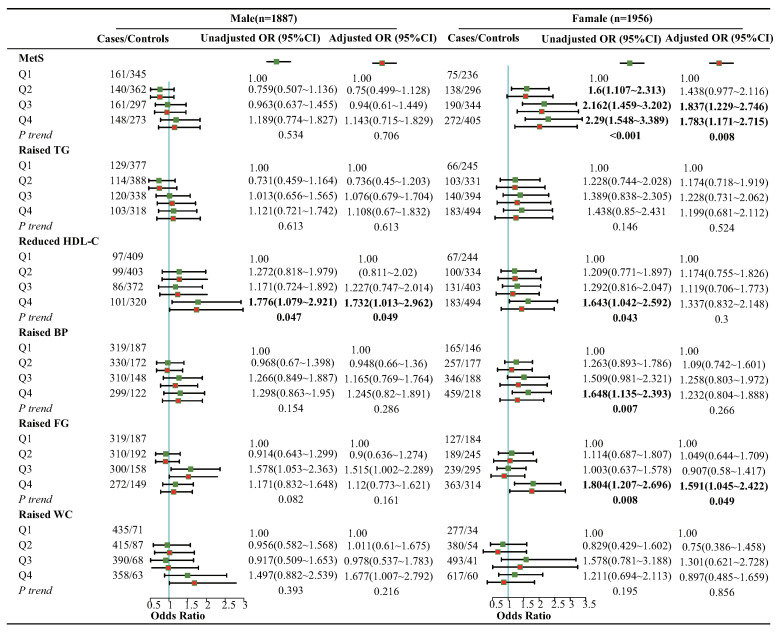
Subgroup analysis of association between DII and MetS as a whole and its components among different gender groups. Adjusted for confounding factors such as age, gender, race, education, poverty–income ratio, smoking, alcohol consumption, and sedentary behavior. Q1 [−5.21, −1.33), Q2 [−1.33, 0.10), Q3 [0.10, 1.52), Q4 [1.52, 4.43) for male; Q1 [−4.71, −0.54), Q2 [−0.54, 1.10), Q3 [1.10, 2.37), Q4 [2.37, 4.66) for female.

**Figure 5:**
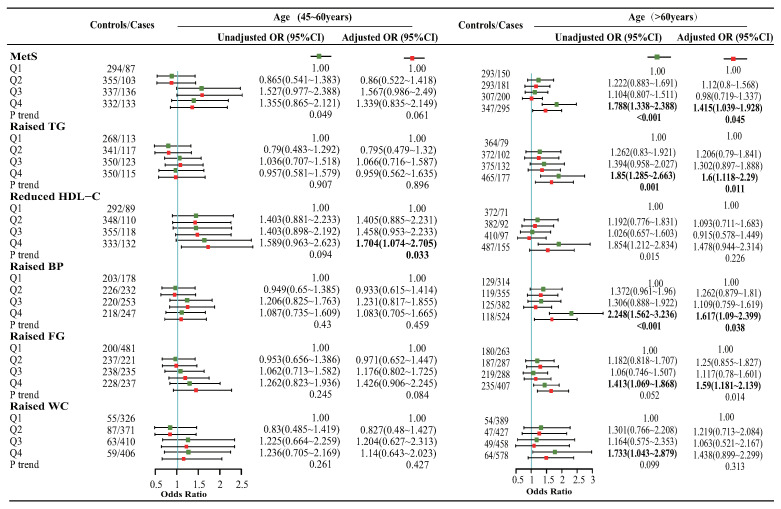
Subgroup analysis of association between DII and MetS as a whole and its components among different age groups. Adjusted for confounding factors such as age, gender, race, education, poverty–income ratio, smoking, alcohol consumption, and sedentary behavior. Q1 [−5.21, −1.10), Q2 [−1.10, 0.60), Q3 [0.60, 2.09), Q4 [2.09, 4.23) for the age range of 45 to 60 years; Q1 [−4.71, −0.89), Q2 [−0.89, 0.66), Q3 [0.66, 1.99), Q4 [1.99, 4.66) for age over 60 years.

To evaluate the potential influence of sex and age on our study, we conducted separate analyses stratified by gender and age (Figure 4 and Figure 5). Our results showed that, after controlling for other factors, the risk of MetS (OR${}_{Q4:Q1}$ = 1.783; 95% CI: 1.171, 2.715; *p*-trend = 0.008) and its component of raised FG (OR${}_{Q4:Q1}$ = 1.591; 95% CI: 1.045, 2.422; *p*-trend = 0.049), increased significantly in the high DII group (Q4) compared to the low DII group (Q1) among females. Meanwhile, the high dietary inflammation group also exhibited an elevated risk of reduced HDL-C (OR${}_{Q4:Q1}$ = 1.732; 95% CI: 1.013, 2.962; *p*-trend = 0.047) among males. In the analysis of different age groups, the highest DII group was associated with an increased risk of MetS (OR${}_{Q4:Q1}$ = 1.415; 95% CI: 1.039, 1.928; *p*-trend = 0.045), and its component of raised TG (OR${}_{Q4:Q1}$ = 1.6; 95% CI: 1.118, 2.29; *p*-trend = 0.011), raised BP (OR${}_{Q4:Q1}$ = 1.617; 95% CI: 1.09, 2.399; *p*-trend = 0.038), and raised FG (OR${}_{Q4:Q1}$ = 1.59; 95% CI: 1.181, 2.139; *p*-trend = 0.014) in individuals aged over 60 years. However, in the 45∼60 age group, a significant association between high DII and reduced HDL-C (OR${}_{Q4:Q1}$ = 1.704; 95% CI: 1.074, 2.705; *p*-trend = 0.033) was observed, while such an association was not observed in the group aged >60 years.

### 3.3. Association of DII Levels with Biochemical Markers of MetS

**Figure 6:**
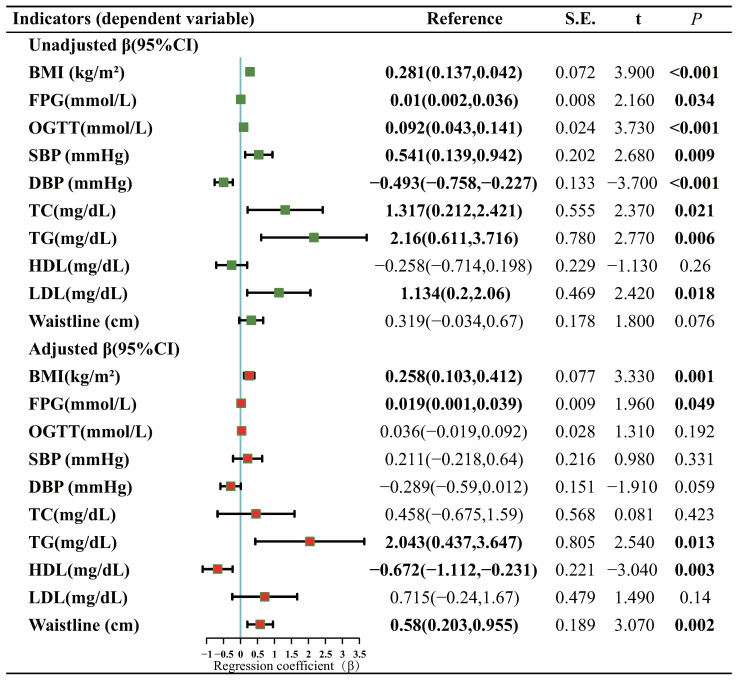
Generalized linear regression analysis of DII levels and indicators of MetS. BMI, body mass index; SBP, systolic blood pressure; DBP, diastolic blood pressure; HDL-C, high-density lipoprotein cholesterol; LDL-C, low-density lipoprotein cholesterol; TC, total cholesterol; TG, total triglycerides; FPG, fasting blood sugar; OGTT, oral glucose tolerance test. Adjusted for confounding factors such as age, gender, race, education, poverty–income ratio, smoking, alcohol consumption, and sedentary behavior. Green: No adjustments were made; Red: Adjusted for confounding factors.

**Figure 7:**
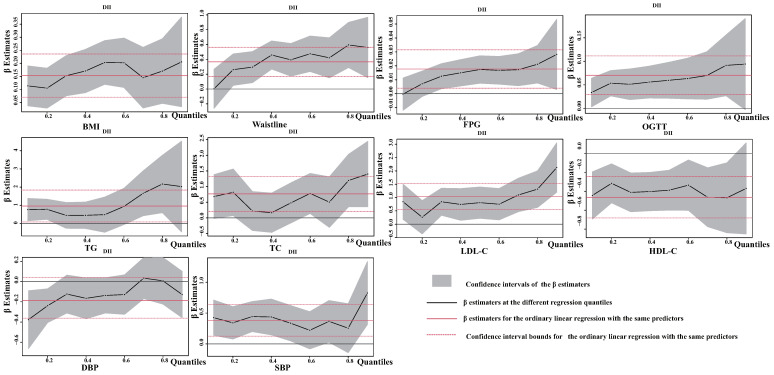
Quantile regression estimation coefficient plot of DII level versus biochemical index of MetS. Adjusted for confounding factors such as age, gender, race, education, poverty–income ratio, smoking, alcohol consumption, and sedentary behavior.

A generalized linear regression model was utilized to establish the correlation between levels of DII and indicators of MetS (Figure 6). Upon adjusting for variables such as age, gender, ethnicity, education, poverty–income ratio, smoking, alcohol consumption, and sedentary time, a statistically significant difference (*p* < 0.05) was found between the DII level and the remaining five metrics, excluding SBP, DBP, OGTT, TC, and LDL-C. Specifically, there was a positive correlation between DII levels and BMI (β = 0.258, *p* = 0.001), FPG (β = 0.019, *p* = 0.049), TG (β = 2.043, *p* = 0.013), waistline (β = 0.580, *p* = 0.002), and a negative correlation with HDL-C (β = −0.672, *p* = 0.003). Additionally, the quantile regression model analyzed the association between MetS-related indicators and various quantile points of the DII level (Figure 7). The results were consistent with those of the generalized linear regression, showing a gradually increasing trend in the positive correlation between DII levels and BMI, waistline, FPG, and TG, and a gradual decrease in the correlation with HDL-C. The findings suggest that a pro-inflammatory diet increases the risk of obesity, hyperglycemia, and hyperlipidemia.

## 4. Discussion

In this study, we investigated the association between DII scores and MetS in middle-aged and older adults using data from the National Health and Nutrition Examination Survey (NHANES). Following adjustment for confounding factors, our study revealed that individuals aged 45 years or older, with high DII scores, were at a greater risk of developing MetS, decreased levels of HDL-C, and elevated FG compared to those with low DII scores. Gender and age-specific analyses revealed that women with high DII scores had a greater likelihood of experiencing MetS and raised FG, while men with high DII scores had an increased risk of reduced HDL-C. Furthermore, DII scores were linked to a greater likelihood of MetS and its constituent components of raised TG, raised BP, and raised FG in individuals aged 60 years and above. Finally, we employed generalized linear regression and quantile regression models to evaluate the correlation between DII and MetS-related markers and found a notable relationship between DII scores and BMI, waist circumference, FPG, TG, and HDL-C levels. Our findings support the use of DII scores as a dietary assessment tool and underscore the importance of dietary interventions in preventing MetS.

Elevated levels of inflammatory markers, including IL-6, C-reactive protein (CRP), and TNF-α, have been associated with higher levels of DII in the bloodstream [31,32]. This suggests that reducing the DII through dietary intervention may prevent the development and progression of MetS. Our findings are consistent with other studies conducted in various countries, including Columbia, Mexico, South Korea, Croatia, China, and Iran [23,33,34,35,36,37]. Previous research has shown that a Western-style diet high in fat, salt, and refined carbohydrates increases the risk of inflammatory diseases, including MetS [38,39]. In contrast, diets with low DII scores, such as the DASH and Mediterranean diets, have been associated with improved health [40,41,42,43]. In addition, various dietary patterns with different DII scores can also affect the composition of the intestinal microbiota and ultimately impact overall health [44]. For instance, a pro-inflammatory diet such as the Western diet is associated with lower gut microbial diversity and reduced probiotic abundance, while an anti-inflammatory diet such as the Mediterranean diet has been found to have higher gut microbial diversity and increased probiotic abundance [45,46]. Therefore, the results indicate that middle-aged and older individuals can prevent and mitigate the onset and progression of MetS by improving their dietary structure. Our study also found that middle-aged and older women with higher DII levels were more prone to MetS and hyperglycemia. This is due to the fact that women are more prone to weight gain physiologically, particularly during menopause when levels of estrogen in the body decrease, leading to increased fat storage. With age, the distribution of fat within the female body also changes, shifting from the lower limbs to the abdominal area, causing an increase in abdominal fat [47,48]. Additionally, insulin sensitivity decreases in women during menopause, leading to weakened glucose control and a slowing of glucose metabolism [49,50]. Studies have also found that women tend to be less physically active than men and are also more susceptible to overeating due to lifestyle stress and emotional factors [51]. These factors all increase the risk of women developing MetS. To prevent the onset of MetS, women need to pay more attention to preventing it by maintaining healthy dietary and exercise habits. Moreover, the study found that high DII scores were associated with an increased risk of MetS in individuals aged 60 years and older, but not in those aged 45 to 60 years. Aging is also associated with chronic low-grade inflammation, which may exacerbate the inflammatory effects of a high DII diet, leading to a higher risk of developing MetS [52,53]. In contrast, individuals aged 45 to 60 years may be more able to control their dietary habits and physical activity levels, which could help to counteract the negative effects of a high DII diet. However, it is important to note that our study only found an association between DII and MetS, and further research is needed to determine causality and to explore the mechanisms behind the observed association.

In addition, in our investigation, we sought to examine the relationship between indicators of MetS and DII scores through both linear and quantile regression models. Results showed that among all MetS indicators, DII was significantly linked with BMI, FPG, TG, HDL-C, and waistline. In a study conducted in East Azerbaijan, Iran, it was found that individuals in the highest quartile of DII scores had significantly elevated levels of FBG (OR = 2.56, 95% CI = 1.00 to 7.05) [23]. Meanwhile, in a Dutch cohort study, it was observed that increasing DII levels were associated with elevated fasting blood glucose concentrations (β = 0.09, 95% CI = 0.01, 0.17) [20]. However, a study conducted in Luxembourg found no correlation between DII and serum glucose levels [19]. The disparities in the correlation between DII scores and MetS indicators may stem from disparities in the study population and variations in the techniques employed for dietary evaluation and the foods taken into account for DII calculation. Studies have shown that high DII diets contain large amounts of processed foods, which are rich in saturated fatty acids and trans-fatty acids and high in sugar, affecting the secretion of gut hormones and digestive enzymes within the intestine, suppressing insulin secretion, and leading to an increase in fat storage in the body [54]. In addition, high DII diets cause an imbalance in gut microbiota, leading to an overgrowth of pathogenic bacteria and the production of large amounts of endotoxins, which enter the circulation system and cause low-grade inflammation, leading to metabolic disturbance in the body [55]. Therefore, a low DII diet pattern is positively impactful on the health of middle-aged and elderly individuals.

To our knowledge, this is the first study to investigate the association between DII scores and MetS-related markers in middle-aged and elderly individuals aged 45 or above in the United States. Despite some limitations, such as its cross-sectional design that impedes any causal inference, possible errors in dietary recall and covariate data due to self-reported information, the likelihood of unknown confounding factors that are not present in the NHANES database, and the potential lack of generalizability to other countries due to inter-country differences, the study results are based on a large, diverse sample of non-institutionalized participants and may be generalizable to the overall U.S. population.

In conclusion, this study’s findings offer evidence for promoting healthy dietary practices among middle-aged and elderly individuals to deter the onset of MetS and provide a theoretical foundation for disease prevention and treatment that encompasses dietary management, specifically for those in the pre-disease stage. Further, in-depth prospective studies are required to establish a causal relationship between an inflammatory diet and the development of MetS and to examine the role of dietary composition in the etiology of the disease. Ultimately, strategies should be devised for the widespread prevention and treatment of chronic illnesses.

## 5. Conclusions

In the United States, among middle-aged and elderly individuals, a high DII score was found to be correlated with the presence of MetS, reduced HDL-C, and raised FG. Therefore, dietary recommendations for the middle-aged and elderly in the United States should focus on reducing the DII by choosing foods rich in antioxidants, dietary fiber, and unsaturated fatty acids, such as vegetables, fruits, nuts, whole grains, deep-sea fish, flaxseeds, etc. Additionally, they should reduce their intake of processed foods, such as high sugar, high salt, high-fat foods, and carbohydrates with high calorie levels.

## Figures and Tables

**Table 1:** DII quartile characteristic distribution of the basic situation of the research subjects [*n* (%) or M(P25, P75)].

Characteristics	All (*n* = 3843)	Q1 (*n* = 817)	Q2 (*n* = 936)	Q3 (*n* = 992)	Q4 (*n* = 1098)	*p*-Value
Range	−5.20∼4.66	−5.20∼−1.00	−1.00∼0.63	0.63∼2.05	2.05∼4.66	
Sex						
Male	1887 (49.1)	506 (61.93)	502 (53.63)	458 (46.17)	421 (38.34)	**<0.001**
Female	1956 (50.9)	311 (38.07)	434 (46.37)	534 (53.83)	677 (61.66)	
Age						
45∼60	1777 (46.24)	403 (49.33)	449 (47.97)	447 (45.06)	478 (43.53)	0.4
≥60	2066 (53.76)	414 (50.67)	487 (52.03)	545 (54.94)	620 (56.47)	
Education						
Less than 9th grade	407 (10.59)	62 (1.58)	91 (9.74)	111 (11.19)	143 (13.02)	**<0.001**
9∼11th grade	499 (12.98)	67 (8.20)	97 (10.36)	136 (13.71)	199 (18.12)	
High school graduate	881 (22.92)	134 (21.22)	199 (21.26)	235 (23.69)	313 (28.51)	
Some college or AA degree	1069 (27.82)	233 (30.23)	283 (30.23)	284 (28.63)	269 (24.50)	
College graduate or above	987 (25.68)	321 (28.42)	266 (28.41)	226 (22.78)	174 (15.85)	
Race						
Mexican American	406 (11.97)	96 (11.75)	132 (14.10)	112 (11.29)	120 (10.93)	**0.006**
Other Hispanic	407 (10.59)	73 (12.70)	92 (9.83)	126 (12.70)	116 (10.56)	
Non-Hispanic White	2039 (53.06)	459 (52.12)	509 (54.38)	517 (52.12)	554 (50.46)	
Non-Hispanic Black	660 (17.17)	103 (17.44)	134 (14.32)	173 (17.44)	250 (22.77)	
Other Race	277 (7.21)	86 (6.45)	69 (7.37)	64 (6.45)	58 (5.28)	
PIR						
<1	619 (16.11)	80 (9.79)	128 (13.68)	180 (18.15)	231 (21.04)	**<0.001**
1∼1.99	966 (25.14)	183 (22.40)	200 (21.37)	242 (24.40)	341 (31.06)	
2∼3.99	1051 (27.35)	175 (21.42)	270 (28.85)	292 (29.44)	314 (28.60)	
≥4	1207 (31.41)	379 (46.39)	338 (36.11)	278 (28.02)	212 (19.31)	
Smoking						
Yes	1917 (49.88)	398 (48.71)	466 (49.79)	493 (49.70)	560 (51.00)	0.8
No	1926 (50.12)	419 (51.29)	470 (50.21)	499 (50.30)	538 (49.00)	
Alcohol						
Yes	2772 (72.13)	643 (78.70)	722 (77.14)	713 (71.88)	694 (63.21)	**<0.001**
No	1071 (27.87)	174 (21.30)	214 (22.86)	279 (28.12)	404 (36.79)	
Sedentary						
<3 h	575 (14.96)	109 (13.34)	137 (14.64)	157 (15.83)	172 (15.66)	0.2
3∼5.9 h	1421 (36.98)	283 (34.64)	359 (38.35)	382 (38.51)	397 (36.16)	
≥6 h	1847 (48.06)	425 (52.02)	440 (47.01)	453 (45.67)	529 (48.18)	
Metabolic syndrome						
Non-Mets	2558 (66.56)	581 (71.11)	658 (70.30)	641 (64.62)	678 (61.75)	**0.005**
Mets	1285 (33.44)	236 (28.89)	278 (29.70)	351 (35.38)	420 (38.25)	
SBP (mmHg)	124 (114,136)	122 (114,134)	122 (112,134)	124 (114,138)	124 (114,136)	**0.04**
DBP (mmHg)	70 (64,78)	70 (62,76)	70 (64,76)	72 (64, 78)	72 (64,78)	**0.01**
TC (mg/dL)	202 (176,230)	199 (173,228)	202 (176,226)	203 (177,234)	203 (178,232)	0.3
TG (mg/dL)	106 (75,151)	103 (72,149)	105 (71,144)	105 (76,158)	111 (81,155)	**0.03**
LDL-C (mg/dL)	120 (98,145)	119 (94,140)	118 (97,145)	120 (98,147)	122 (103, 146)	0.5
HDL-C (mg/dL)	54 (45,67)	53 (45,65)	55 (46,68)	54 (46,66)	53 (44,67)	0.4
FPG (mmol/L)	5.61 (5.27,6.05)	5.60 (5.27,6.00)	5.60 (5.25,5.99)	5.66 (5.22,6.05)	5.66 (5.32,6.10)	0.3
OGTT (mmol/L)	6.43 (5.16,8.10)	6.27 (5.11,7.88)	6.27 (5.16,7.88)	6.38 (5.11,8.05)	6.71 (5.32,8.54)	**0.002**
BMI (kg/m${}^{2}$)	27.6 (24.4,31.6)	26.9 (24.0,30.8)	27.3 (24.3,31.3)	27.8 (24.7,31.9)	28.1 (24.9,32.8)	**0.009**
Waistline (cm)	98.9 (90.1,108.9)	98.5 (89.9,108.2)	98.3 (90.3,108.4)	98.4 (89.0,108.3)	100.0 (90.9,110.4)	0.4

BMI, body mass index; SBP, systolic blood pressure; DBP, diastolic blood pressure; HDL-C, high-density lipoprotein cholesterol; LDL-C, Low-density lipoprotein cholesterol; TC, total cholesterol; TG, total triglycerides; FPG, fasting blood sugar; OGTT, oral glucose tolerance test; PIR, poverty–income ratio. Data are presented as *n* (%) for categorical data, medians (interquartile ranges) for nonparametrically distributed data. Variables with *p*-values less than 0.05 were highlighted in bold font.

## Data Availability

The original data for the conclusion of this paper can be obtained from the National Health and Nutrition Examination Survey (NHANES) website (https://www.cdc.gov/nchs/nhanes/index.htm (accessed on 6 June 2022)) or by contacting the author.

## Supplementary Materials

The following supporting information can be downloaded at: https://www.mdpi.com/article/10.3390/nu15081857/s1, The supplementary file contains the raw data for this study.
